# Bis(μ-3-chloro­benzene-1,2-dicarboxyl­ato-κ^2^ *O* ^2^:*O* ^2^)bis­[diaqua­(5,5′-dimethyl-2,2′-bipyridine-κ^2^ *N*,*N*′)copper(II)]

**DOI:** 10.1107/S1600536811035112

**Published:** 2011-09-14

**Authors:** Fu-An Li, Fu Xu, Xiao-Ming Hu

**Affiliations:** a College of Chemistry and Chemical Engineering, Pingdingshan University, Pingdingshan 467000, Henan, People’s Republic of China

## Abstract

In the centrosymmetric binuclear title compound, [Cu_2_(C_8_H_3_ClO_4_)_2_(C_12_H_12_N_2_)_2_(H_2_O)_4_], the Cu^II^ ion is six-coordinated by two N atoms from a 5,5′-dimethyl-2,2′-bipyridine ligand, two bridging O atoms from two 3-chloro­benzene-1,2-dicarboxyl­ate ligands and two water mol­ecules in a distorted octa­hedral geometry. The binuclear complex mol­ecules are linked together by inter­molecular O—H⋯O hydrogen bonds into a layer parallel to (100). The layers are connected by C—H⋯Cl hydrogen bonds. Intra­molecular O—H⋯O hydrogen bonds and π–π inter­actions [centroid–centroid distance = 3.5958 (16) Å] are also present.

## Related literature

For background to polynuclear coordination compounds containing benzene­carboxyl­ate ligands, see: Baca *et al.* (2005 ▶); Ma *et al.* (2004 ▶); Thirumurugan & Rao (2005 ▶); Zang *et al.* (2010 ▶). For O—H⋯O and C—H⋯Cl hydrogen bonds, see: Desiraju (2004 ▶); Song & Iyoda (2009 ▶); Wang *et al.* (2011 ▶).
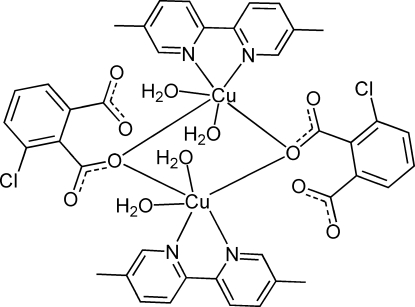


## Experimental

### 

#### Crystal data

[Cu_2_(C_8_H_3_ClO_4_)_2_(C_12_H_12_N_2_)_2_(H_2_O)_4_]
*M* *_r_* = 964.72
Monoclinic, 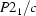

*a* = 11.6908 (7) Å
*b* = 11.8643 (6) Å
*c* = 17.2869 (13) Å
β = 124.806 (5)°
*V* = 1968.8 (2) Å^3^
*Z* = 2
Mo *K*α radiation
μ = 1.29 mm^−1^
*T* = 296 K
0.21 × 0.20 × 0.19 mm

#### Data collection

Bruker APEXII CCD diffractometer
Absorption correction: multi-scan (*SADABS*; Bruker, 2001 ▶) *T*_min_ = 0.774, *T*_max_ = 0.792
7602 measured reflections
3450 independent reflections
2845 reflections with *I* > 2σ(*I*)
*R*_int_ = 0.030

#### Refinement

*R*[*F* ^2^ > 2σ(*F* ^2^)] = 0.036
*wR*(*F* ^2^) = 0.094
*S* = 1.07
3450 reflections
273 parameters
H-atom parameters constrained
Δρ_max_ = 0.34 e Å^−3^
Δρ_min_ = −0.29 e Å^−3^

### 

Data collection: *APEX2* (Bruker, 2007 ▶); cell refinement: *SAINT* (Bruker, 2007 ▶); data reduction: *SAINT*; program(s) used to solve structure: *SHELXTL* (Sheldrick, 2008 ▶); program(s) used to refine structure: *SHELXTL*; molecular graphics: *DIAMOND* (Brandenburg, 1999 ▶); software used to prepare material for publication: *SHELXTL*.

## Supplementary Material

Crystal structure: contains datablock(s) I, global. DOI: 10.1107/S1600536811035112/hy2462sup1.cif

Structure factors: contains datablock(s) I. DOI: 10.1107/S1600536811035112/hy2462Isup2.hkl

Additional supplementary materials:  crystallographic information; 3D view; checkCIF report

## Figures and Tables

**Table 1:** Hydrogen-bond geometry (Å, °)

*D*—H⋯*A*	*D*—H	H⋯*A*	*D*⋯*A*	*D*—H⋯*A*
O1*W*—H1*WA*⋯O3	0.85	1.79	2.622 (3)	164
O1*W*—H1*WB*⋯O4^i^	0.85	1.82	2.655 (3)	167
O2*W*—H2*WA*⋯O1	0.85	2.17	2.731 (3)	124
O2*W*—H2*WB*⋯O4^i^	0.85	2.06	2.786 (3)	143
C6—H6⋯Cl1^ii^	0.93	2.82	3.609 (4)	144

Symmetry codes: (i) 
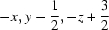
; (ii) 
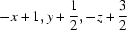
.

**Fig. 1.**
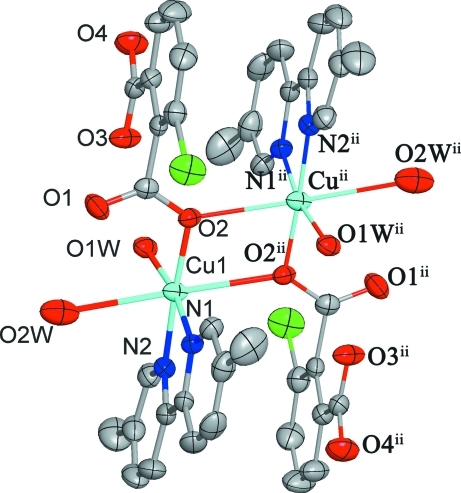
Molecular structure of the title compound. Displacement ellipsoids are drawn at the 50% probability level. H atoms are omitted for clarity. [Symmetry code: (i) -x, 2-y, 1-z.]

**Fig. 2.**
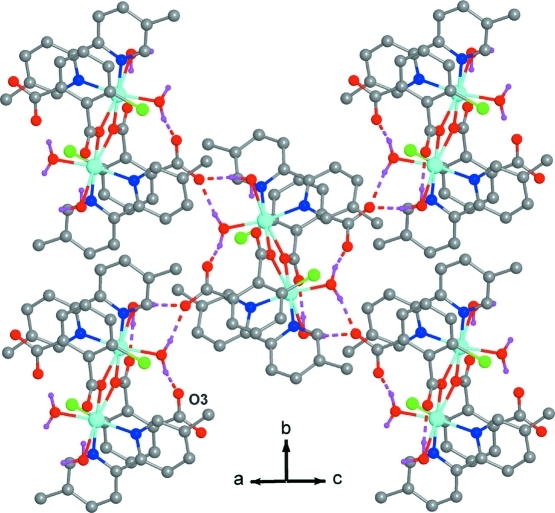
A view of the supramolecular layer in the title compound. Dotted lines represent hydrogen bonds.

**Fig. 3.**
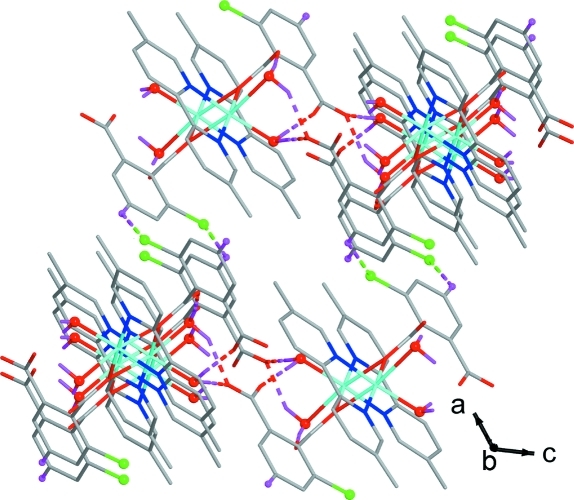
The three-dimensional supramolecular structure in the title compound. Dashed lines indicate hydrogen bonds.

## supplementary crystallographic information

### Comment

It is common knowledge that the coordination geometry of metal ion and the shape and bonding mode of ligand are generally the primary considerations in metal-mediated self-assembly reactions. Relatively small changes in the bridging ligand can give rise to large variation in the overall structure of the assembly. Recently, some polynuclear coordination compounds containing benzenecarboxylate ligands and O—H···O and C—H···Cl hydrogen bonds (Desiraju, 2004; Song & Iyoda, 2009; Wang *et al.*, 2011) have been reported (Baca *et al.*, 2005; Ma *et al.*, 2004; Thirumurugan & Rao, 2005; Zang *et al.*, 2010). To better understand the influence of benzenecarboxylate ligands and hydrogen-bonding interactions on the resultant structures, we have begun working on the architectures of polynuclear structures from 3-chlorobenzene-1,2-dioic acid. As part of our ongoing investigation, the title compound has been prepared and its structure was determined.

The title compound is a binuclear complex (Fig. 1). The Cu^II^ atom is coordinated by two N atoms from a 5,5'-dimethyl-2,2'-bipyridine ligand, two O atoms from two 3-chlorobenzene-1,2-dicarboxylate ligands and two O atoms from two coordinated water molecules, forming a distorted octahedral geometry. As shown in Fig. 2, each complex molecule is connected to four neighboring molecules through O—H···O hydrogen bonds (Table 1), resulting in a two-dimensional supramolecular structure parallel to (1 0 0). Adjacent layers are associated together by C—H···Cl hydrogen bonds, forming a three-dimensional supramolecular structure (Fig. 3).

### Experimental

A mixture of CuSO_4_.5H_2_O (7.5 mg, 0.03 mmol), 3-chlorobenzene-1,2-dioic acid (6 mg, 0.03 mmol), 5,5'-dimethyl-2,2'-bipyridine (5.5 mg, 0.03 mmol) and NaOH (2.4 mg, 0.06 mmol) in 10 ml of H_2_O was sealed in a stainless-steel reactor with a Teflon liner and heated at 393 K for 72 h. A quantity of green single crystals was obtained after the solution was cooled to room temperature at a rate of 10 K h^-1^.

### Refinement

H atoms were positioned geometrically and refined using a riding model, with C—H = 0.93 (aromatic) and 0.96 (methyl) Å and with *U*_iso_(H) = 1.2(1.5 for methyl)*U*_eq_(C). H atoms of the water molecules were located from a difference Fourier map and refined with a distance restraint of O—H = 0.85 Å and with *U*_iso_(H) = 1.5*U*_eq_(O).

### Crystal data

**Table:**

[Cu_2_(C_8_H_3_ClO_4_)_2_(C_12_H_12_N_2_)_2_(H_2_O)_4_]	*F*(000) = 988
*M**_r_* = 964.72	*D*_x_ = 1.627 Mg m^−^^3^
Monoclinic, *P*2_1_/*c*	Mo *K*α radiation, λ = 0.71073 Å
Hall symbol: -P 2ybc	Cell parameters from 2332 reflections
*a* = 11.6908 (7) Å	θ = 3.0–29.3°
*b* = 11.8643 (6) Å	µ = 1.29 mm^−^^1^
*c* = 17.2869 (13) Å	*T* = 296 K
β = 124.806 (5)°	Block, green
*V* = 1968.8 (2) Å^3^	0.21 × 0.20 × 0.19 mm
*Z* = 2	

### Data collection

**Table:**

Bruker APEXII CCD diffractometer	3450 independent reflections
Radiation source: fine-focus sealed tube	2845 reflections with *I* > 2σ(*I*)
graphite	*R*_int_ = 0.030
φ and ω scans	θ_max_ = 25.0°, θ_min_ = 3.0°
Absorption correction: multi-scan (*SADABS*; Bruker, 2001)	*h* = −13→12
*T*_min_ = 0.774, *T*_max_ = 0.792	*k* = −13→14
7602 measured reflections	*l* = −19→20

### Refinement

**Table:**

Refinement on *F*^2^	Primary atom site location: structure-invariant direct methods
Least-squares matrix: full	Secondary atom site location: difference Fourier map
*R*[*F*^2^ > 2σ(*F*^2^)] = 0.036	Hydrogen site location: inferred from neighbouring sites
*wR*(*F*^2^) = 0.094	H-atom parameters constrained
*S* = 1.07	*w* = 1/[σ^2^(*F*_o_^2^) + (0.0451*P*)^2^] where *P* = (*F*_o_^2^ + 2*F*_c_^2^)/3
3450 reflections	(Δ/σ)_max_ = 0.001
273 parameters	Δρ_max_ = 0.34 e Å^−^^3^
0 restraints	Δρ_min_ = −0.29 e Å^−^^3^

### Fractional atomic coordinates and isotropic or equivalent isotropic displacement parameters (Å^2^)

**Table:**

	*x*	*y*	*z*	*U*_iso_*/*U*_eq_	
Cu1	0.00030 (3)	0.85763 (3)	0.53560 (2)	0.02829 (13)	
O1	0.2700 (2)	0.90830 (15)	0.72939 (15)	0.0422 (5)	
O2	0.11583 (17)	0.99350 (14)	0.59290 (12)	0.0273 (4)	
O3	0.0506 (2)	1.03936 (16)	0.73724 (14)	0.0417 (5)	
O4	0.0696 (2)	1.19348 (17)	0.81467 (15)	0.0481 (6)	
O1W	−0.08536 (18)	0.88808 (15)	0.60396 (13)	0.0323 (4)	
H1WA	−0.0475	0.9459	0.6386	0.048*	
H1WB	−0.0733	0.8317	0.6382	0.048*	
O2W	0.1570 (2)	0.69797 (18)	0.67213 (17)	0.0595 (7)	
H2WA	0.2298	0.7286	0.7187	0.089*	
H2WB	0.1093	0.6695	0.6903	0.089*	
N1	0.0910 (2)	0.80054 (17)	0.47615 (15)	0.0283 (5)	
N2	−0.1213 (2)	0.72318 (17)	0.47124 (15)	0.0291 (5)	
C1	0.2153 (3)	0.9926 (2)	0.68096 (18)	0.0275 (6)	
C2	0.1059 (3)	1.1301 (2)	0.77408 (19)	0.0294 (6)	
C3	0.2744 (3)	1.1063 (2)	0.72625 (18)	0.0262 (6)	
C4	0.2292 (3)	1.1679 (2)	0.77316 (19)	0.0313 (7)	
C5	0.2972 (3)	1.2677 (2)	0.8182 (2)	0.0442 (8)	
H5	0.2670	1.3091	0.8490	0.053*	
C6	0.4079 (4)	1.3067 (3)	0.8183 (2)	0.0535 (9)	
H6	0.4529	1.3728	0.8501	0.064*	
C7	0.4520 (3)	1.2485 (3)	0.7716 (2)	0.0438 (8)	
H7	0.5257	1.2751	0.7704	0.053*	
C8	0.3848 (3)	1.1490 (2)	0.7262 (2)	0.0332 (7)	
C9	0.1929 (3)	0.8511 (2)	0.47533 (19)	0.0329 (7)	
H9	0.2267	0.9196	0.5066	0.040*	
C10	0.2500 (3)	0.8080 (3)	0.4313 (2)	0.0430 (8)	
C11	0.3573 (4)	0.8730 (3)	0.4275 (3)	0.0691 (11)	
H11A	0.3784	0.9420	0.4621	0.104*	
H11B	0.3214	0.8898	0.3631	0.104*	
H11C	0.4405	0.8287	0.4548	0.104*	
C12	0.2011 (3)	0.7028 (3)	0.3883 (2)	0.0457 (8)	
H12	0.2390	0.6687	0.3593	0.055*	
C13	0.0978 (3)	0.6501 (2)	0.3887 (2)	0.0432 (8)	
H13	0.0651	0.5803	0.3599	0.052*	
C14	0.0421 (3)	0.7003 (2)	0.43179 (18)	0.0290 (6)	
C15	−0.0748 (3)	0.6550 (2)	0.43229 (19)	0.0317 (7)	
C16	−0.1362 (3)	0.5512 (2)	0.3958 (2)	0.0435 (8)	
H16	−0.1047	0.5051	0.3682	0.052*	
C17	−0.2437 (3)	0.5169 (2)	0.4008 (2)	0.0482 (9)	
H17	−0.2842	0.4467	0.3772	0.058*	
C18	−0.2924 (3)	0.5854 (2)	0.4403 (2)	0.0398 (7)	
C19	−0.4090 (3)	0.5521 (3)	0.4481 (3)	0.0620 (10)	
H19A	−0.4961	0.5765	0.3929	0.093*	
H19B	−0.3959	0.5867	0.5028	0.093*	
H19C	−0.4099	0.4716	0.4536	0.093*	
C20	−0.2279 (3)	0.6894 (2)	0.4739 (2)	0.0353 (7)	
H20	−0.2605	0.7379	0.4995	0.042*	
Cl1	0.44008 (8)	1.07793 (7)	0.66549 (6)	0.0534 (2)	

### Atomic displacement parameters (Å^2^)

**Table:**

	*U*^11^	*U*^22^	*U*^33^	*U*^12^	*U*^13^	*U*^23^
Cu1	0.0343 (2)	0.0238 (2)	0.0340 (2)	−0.00589 (14)	0.02376 (18)	−0.00736 (15)
O1	0.0441 (12)	0.0252 (10)	0.0467 (13)	0.0041 (10)	0.0196 (10)	0.0063 (10)
O2	0.0310 (10)	0.0261 (9)	0.0260 (10)	−0.0061 (8)	0.0170 (9)	−0.0060 (9)
O3	0.0569 (13)	0.0368 (11)	0.0489 (13)	−0.0175 (10)	0.0405 (11)	−0.0177 (10)
O4	0.0625 (14)	0.0416 (12)	0.0574 (15)	0.0008 (11)	0.0444 (13)	−0.0117 (11)
O1W	0.0441 (11)	0.0252 (9)	0.0366 (11)	−0.0045 (9)	0.0284 (10)	−0.0031 (9)
O2W	0.0635 (15)	0.0554 (14)	0.0714 (17)	−0.0126 (12)	0.0455 (14)	−0.0206 (13)
N1	0.0357 (13)	0.0248 (12)	0.0271 (12)	0.0010 (10)	0.0194 (11)	−0.0024 (10)
N2	0.0336 (13)	0.0242 (11)	0.0271 (12)	−0.0014 (10)	0.0159 (11)	0.0003 (10)
C1	0.0281 (14)	0.0294 (14)	0.0321 (16)	−0.0001 (12)	0.0213 (13)	−0.0032 (13)
C2	0.0386 (16)	0.0265 (14)	0.0246 (14)	0.0025 (13)	0.0190 (13)	0.0014 (12)
C3	0.0278 (14)	0.0233 (13)	0.0202 (13)	−0.0004 (12)	0.0094 (12)	0.0033 (12)
C4	0.0399 (16)	0.0258 (13)	0.0260 (15)	−0.0024 (13)	0.0175 (13)	−0.0008 (13)
C5	0.062 (2)	0.0355 (16)	0.0428 (19)	−0.0137 (16)	0.0344 (17)	−0.0129 (15)
C6	0.073 (2)	0.0365 (17)	0.056 (2)	−0.0237 (17)	0.040 (2)	−0.0156 (17)
C7	0.0390 (16)	0.0455 (18)	0.0418 (19)	−0.0152 (15)	0.0200 (15)	−0.0018 (16)
C8	0.0296 (15)	0.0365 (15)	0.0323 (16)	0.0012 (13)	0.0170 (13)	0.0029 (14)
C9	0.0379 (16)	0.0341 (15)	0.0286 (15)	0.0016 (13)	0.0200 (14)	−0.0002 (13)
C10	0.0429 (18)	0.057 (2)	0.0355 (17)	0.0078 (16)	0.0265 (15)	0.0077 (16)
C11	0.066 (2)	0.099 (3)	0.069 (3)	−0.013 (2)	0.055 (2)	−0.007 (2)
C12	0.056 (2)	0.056 (2)	0.0368 (18)	0.0155 (17)	0.0341 (17)	0.0012 (16)
C13	0.061 (2)	0.0343 (16)	0.0388 (18)	0.0077 (15)	0.0313 (17)	−0.0042 (15)
C14	0.0376 (16)	0.0250 (14)	0.0227 (14)	0.0053 (12)	0.0162 (13)	0.0025 (12)
C15	0.0393 (17)	0.0251 (14)	0.0233 (14)	0.0022 (13)	0.0134 (13)	0.0021 (12)
C16	0.057 (2)	0.0276 (15)	0.0389 (18)	−0.0044 (15)	0.0233 (16)	−0.0087 (14)
C17	0.050 (2)	0.0288 (15)	0.0441 (19)	−0.0129 (15)	0.0141 (16)	−0.0029 (15)
C18	0.0358 (16)	0.0363 (16)	0.0326 (16)	−0.0078 (14)	0.0109 (14)	0.0044 (15)
C19	0.050 (2)	0.058 (2)	0.067 (2)	−0.0219 (18)	0.0273 (19)	−0.002 (2)
C20	0.0314 (15)	0.0360 (15)	0.0314 (16)	−0.0018 (13)	0.0138 (13)	0.0011 (14)
Cl1	0.0504 (5)	0.0573 (5)	0.0724 (6)	−0.0032 (4)	0.0468 (5)	−0.0078 (5)

### Geometric parameters (Å, °)

**Table:**

Cu1—O2	1.9689 (16)	C7—C8	1.387 (4)
Cu1—O2^i^	2.5406 (17)	C7—H7	0.9300
Cu1—N1	1.971 (2)	C8—Cl1	1.733 (3)
Cu1—N2	2.000 (2)	C9—C10	1.366 (4)
Cu1—O1W	1.9700 (19)	C9—H9	0.9300
Cu1—O2W	2.753 (2)	C10—C12	1.397 (4)
O1—C1	1.224 (3)	C10—C11	1.506 (4)
O2—C1	1.284 (3)	C11—H11A	0.9600
O3—C2	1.230 (3)	C11—H11B	0.9600
O4—C2	1.256 (3)	C11—H11C	0.9600
O1W—H1WA	0.8500	C12—C13	1.363 (4)
O1W—H1WB	0.8500	C12—H12	0.9300
O2W—H2WA	0.8500	C13—C14	1.373 (4)
O2W—H2WB	0.8500	C13—H13	0.9300
N1—C9	1.341 (3)	C14—C15	1.474 (4)
N1—C14	1.352 (3)	C15—C16	1.382 (4)
N2—C20	1.334 (3)	C16—C17	1.370 (4)
N2—C15	1.350 (4)	C16—H16	0.9300
C1—C3	1.514 (4)	C17—C18	1.376 (4)
C2—C4	1.518 (4)	C17—H17	0.9300
C3—C8	1.387 (4)	C18—C20	1.389 (4)
C3—C4	1.400 (4)	C18—C19	1.498 (4)
C4—C5	1.390 (4)	C19—H19A	0.9600
C5—C6	1.373 (4)	C19—H19B	0.9600
C5—H5	0.9300	C19—H19C	0.9600
C6—C7	1.367 (5)	C20—H20	0.9300
C6—H6	0.9300		
			
O2—Cu1—O1W	89.18 (7)	C6—C7—H7	120.6
O2—Cu1—N1	97.18 (8)	C8—C7—H7	120.6
O1W—Cu1—N1	170.06 (8)	C7—C8—C3	122.5 (3)
O2—Cu1—N2	177.20 (8)	C7—C8—Cl1	118.1 (2)
O1W—Cu1—N2	92.09 (9)	C3—C8—Cl1	119.4 (2)
N1—Cu1—N2	81.90 (9)	N1—C9—C10	123.9 (3)
O2—Cu1—O2W	101.68 (7)	N1—C9—H9	118.1
O1W—Cu1—O2W	85.94 (7)	C10—C9—H9	118.1
N1—Cu1—O2W	85.29 (8)	C9—C10—C12	116.7 (3)
N2—Cu1—O2W	80.91 (8)	C9—C10—C11	121.1 (3)
O1W—Cu1—O2^i^	101.31 (7)	C12—C10—C11	122.1 (3)
N1—Cu1—O2^i^	87.77 (8)	C10—C11—H11A	109.5
O2—Cu1—O2^i^	75.02 (7)	C10—C11—H11B	109.5
O2W—Cu1—O2^i^	171.88 (8)	H11A—C11—H11B	109.5
N2—Cu1—O2^i^	102.28 (7)	C10—C11—H11C	109.5
C1—O2—Cu1	119.12 (17)	H11A—C11—H11C	109.5
Cu1—O1W—H1WA	109.4	H11B—C11—H11C	109.5
Cu1—O1W—H1WB	109.5	C13—C12—C10	120.1 (3)
H1WA—O1W—H1WB	109.5	C13—C12—H12	120.0
Cu1—O2W—H2WA	109.5	C10—C12—H12	120.0
Cu1—O2W—H2WB	109.5	C12—C13—C14	120.0 (3)
H2WA—O2W—H2WB	109.5	C12—C13—H13	120.0
C9—N1—C14	118.6 (2)	C14—C13—H13	120.0
C9—N1—Cu1	126.69 (18)	N1—C14—C13	120.7 (3)
C14—N1—Cu1	114.75 (18)	N1—C14—C15	114.3 (2)
C20—N2—C15	119.2 (2)	C13—C14—C15	125.0 (3)
C20—N2—Cu1	127.0 (2)	N2—C15—C16	120.6 (3)
C15—N2—Cu1	113.24 (18)	N2—C15—C14	115.1 (2)
O1—C1—O2	125.7 (2)	C16—C15—C14	124.3 (3)
O1—C1—C3	117.9 (2)	C17—C16—C15	119.5 (3)
O2—C1—C3	116.3 (2)	C17—C16—H16	120.3
O3—C2—O4	124.6 (3)	C15—C16—H16	120.3
O3—C2—C4	118.4 (2)	C16—C17—C18	120.7 (3)
O4—C2—C4	117.0 (2)	C16—C17—H17	119.7
C8—C3—C4	118.0 (2)	C18—C17—H17	119.7
C8—C3—C1	118.4 (2)	C17—C18—C20	116.9 (3)
C4—C3—C1	123.6 (2)	C17—C18—C19	123.0 (3)
C5—C4—C3	119.0 (3)	C20—C18—C19	120.1 (3)
C5—C4—C2	119.3 (3)	C18—C19—H19A	109.5
C3—C4—C2	121.7 (2)	C18—C19—H19B	109.5
C6—C5—C4	121.7 (3)	H19A—C19—H19B	109.5
C6—C5—H5	119.2	C18—C19—H19C	109.5
C4—C5—H5	119.2	H19A—C19—H19C	109.5
C7—C6—C5	120.1 (3)	H19B—C19—H19C	109.5
C7—C6—H6	120.0	N2—C20—C18	123.1 (3)
C5—C6—H6	120.0	N2—C20—H20	118.4
C6—C7—C8	118.8 (3)	C18—C20—H20	118.4
			
O1W—Cu1—O2—C1	71.57 (19)	C4—C3—C8—C7	0.9 (4)
N1—Cu1—O2—C1	−100.76 (19)	C1—C3—C8—C7	−175.4 (3)
O2W—Cu1—O2—C1	−14.12 (19)	C4—C3—C8—Cl1	−177.8 (2)
O2—Cu1—N1—C9	−3.7 (2)	C1—C3—C8—Cl1	5.9 (3)
N2—Cu1—N1—C9	173.6 (2)	C14—N1—C9—C10	0.7 (4)
O2W—Cu1—N1—C9	−104.9 (2)	Cu1—N1—C9—C10	−178.8 (2)
O2—Cu1—N1—C14	176.77 (18)	N1—C9—C10—C12	−2.5 (4)
N2—Cu1—N1—C14	−5.90 (18)	N1—C9—C10—C11	175.7 (3)
O2W—Cu1—N1—C14	75.57 (18)	C9—C10—C12—C13	2.2 (5)
O1W—Cu1—N2—C20	7.1 (2)	C11—C10—C12—C13	−176.0 (3)
N1—Cu1—N2—C20	179.1 (2)	C10—C12—C13—C14	−0.2 (5)
O2W—Cu1—N2—C20	92.6 (2)	C9—N1—C14—C13	1.5 (4)
O1W—Cu1—N2—C15	−163.93 (18)	Cu1—N1—C14—C13	−178.9 (2)
N1—Cu1—N2—C15	8.11 (18)	C9—N1—C14—C15	−176.8 (2)
O2W—Cu1—N2—C15	−78.38 (18)	Cu1—N1—C14—C15	2.8 (3)
Cu1—O2—C1—O1	19.6 (4)	C12—C13—C14—N1	−1.7 (4)
Cu1—O2—C1—C3	−164.18 (17)	C12—C13—C14—C15	176.3 (3)
O1—C1—C3—C8	86.6 (3)	C20—N2—C15—C16	−0.2 (4)
O2—C1—C3—C8	−89.9 (3)	Cu1—N2—C15—C16	171.6 (2)
O1—C1—C3—C4	−89.5 (3)	C20—N2—C15—C14	179.4 (2)
O2—C1—C3—C4	94.0 (3)	Cu1—N2—C15—C14	−8.8 (3)
C8—C3—C4—C5	−0.7 (4)	N1—C14—C15—N2	4.1 (3)
C1—C3—C4—C5	175.4 (3)	C13—C14—C15—N2	−174.0 (3)
C8—C3—C4—C2	177.8 (2)	N1—C14—C15—C16	−176.3 (3)
C1—C3—C4—C2	−6.1 (4)	C13—C14—C15—C16	5.5 (4)
O3—C2—C4—C5	−177.1 (3)	N2—C15—C16—C17	−1.0 (4)
O4—C2—C4—C5	1.4 (4)	C14—C15—C16—C17	179.4 (3)
O3—C2—C4—C3	4.5 (4)	C15—C16—C17—C18	1.0 (4)
O4—C2—C4—C3	−177.1 (3)	C16—C17—C18—C20	0.2 (4)
C3—C4—C5—C6	−0.5 (5)	C16—C17—C18—C19	−179.7 (3)
C2—C4—C5—C6	−179.0 (3)	C15—N2—C20—C18	1.5 (4)
C4—C5—C6—C7	1.4 (5)	Cu1—N2—C20—C18	−169.0 (2)
C5—C6—C7—C8	−1.2 (5)	C17—C18—C20—N2	−1.5 (4)
C6—C7—C8—C3	0.0 (5)	C19—C18—C20—N2	178.3 (3)
C6—C7—C8—Cl1	178.7 (3)		

Symmetry codes: (i) −*x*, −*y*+2, −*z*+1.

### Hydrogen-bond geometry (Å, °)

**Table:**

*D*—H···*A*	*D*—H	H···*A*	*D*···*A*	*D*—H···*A*
O1W—H1WA···O3	0.85	1.79	2.622 (3)	164
O1W—H1WB···O4^ii^	0.85	1.82	2.655 (3)	167
O2W—H2WA···O1	0.85	2.17	2.731 (3)	124
O2W—H2WB···O4^ii^	0.85	2.06	2.786 (3)	143
C6—H6···Cl1^iii^	0.93	2.82	3.609 (4)	144

Symmetry codes: (ii) −*x*, *y*−1/2, −*z*+3/2; (iii) −*x*+1, *y*+1/2, −*z*+3/2.

## References

[bb1] Baca, S. G., Filippova, I. G., Ambrus, C., Gdaniec, M., Simonov, Y. A., Gerbeleu, N., Gherco, O. A. & Decurtins, S. (2005). *Eur. J. Inorg. Chem.* pp. 3118–3130.

[bb2] Brandenburg, K. (1999). *DIAMOND* Crystal Impact GbR, Bonn, Germany.

[bb3] Bruker (2001). *SADABS* Bruker AXS Inc., Madison, Wisconsin, USA.

[bb4] Bruker (2007). *APEX2* and *SAINT* Bruker AXS Inc., Madison, Wisconsin, USA.

[bb5] Desiraju, G. R. (2004). *Hydrogen Bonding. Encyclopedia of Supramolecular Chemistry*, edited by J. L. Atwood & J. W. Steed, pp. 658–665. New York: Marcel Dekker Inc.

[bb6] Ma, C.-B., Wang, W.-G., Zhang, X.-F., Chen, C.-N., Liu, Q.-T., Zhu, H.-P., Liao, D.-Z. & Li, L.-C. (2004). *Eur. J. Inorg. Chem.* pp. 3522–3532.

[bb7] Sheldrick, G. M. (2008). *Acta Cryst.* A**64**, 112–122. 10.1107/S0108767307043930 18156677

[bb8] Song, L. J. & Iyoda, T. (2009). *J. Inorg. Organomet. Polym.* **19**, 124–132.

[bb9] Thirumurugan, A. & Rao, C. N. R. (2005). *J. Mater. Chem.* **15**, 3852–3858.

[bb10] Wang, X.-S., Li, Q., Wu, J.-R. & Zhang, M.-M. (2011). *J. Chem. Crystallogr.* **41**, 59–63.

[bb11] Zang, S.-Q., Li, J.-B., Li, Q.-Y., Hou, H.-W. & Mak, T. C. W. (2010). *Polyhedron*, **29**, 2907–2915.

